# The Abuse Potential of Novel Synthetic Phencyclidine Derivative 1-(1-(4-Fluorophenyl)Cyclohexyl)Piperidine (4′-F-PCP) in Rodents

**DOI:** 10.3390/ijms21134631

**Published:** 2020-06-29

**Authors:** In Soo Ryu, Oc-Hee Kim, Young Eun Lee, Ji Sun Kim, Zhan-Hui Li, Tae Wan Kim, Ri-Na Lim, Young Ju Lee, Jae Hoon Cheong, Hee Jin Kim, Yong Sup Lee, Scott C. Steffensen, Bong Hyo Lee, Joung-Wook Seo, Eun Young Jang

**Affiliations:** 1 Pharmacology and Drug Abuse Research Group, Korea Institute of Toxicology, Daejeon 34114, Korea; insoo.ryu@kitox.re.kr (I.S.R.); ochee.kim@kitox.re.kr (O.-H.K.); sherlocked17@naver.com (Y.E.L.); jisun.kim@kitox.re.kr (J.S.K.); lizhanhui1116@163.com (Z.-H.L.); paloma@kitox.re.kr (T.W.K.); rnlim@kitox.re.kr (R.-N.L.); youngju.lee@kitox.re.kr (Y.J.L.); 2 Uimyung Research Institute for Neuroscience, School of Pharmacy, Sahmyook University, Seoul 01795, Korea; cheongjh@syu.ac.kr (J.H.C.); hjkim@syu.ac.kr (H.J.K.); 3 Department of Life and Nanopharmaceutical Sciences, College of Pharmacy, Kyung Hee University, Seoul 02447, Korea; kyslee@khu.ac.kr; 4 Department of Psychology and Neuroscience, Brigham Young University, Provo, UT 84602, USA; scott_steffensen@byu.edu; 5 Department of Acupuncture, Moxibustion and Acupoint, College of Korean Medicine, Daegu Haany University, Daegu 42158, Korea; dlqhdgy@dhu.ac.kr

**Keywords:** abuse potential, conditioned place preference, designer drugs, self-administration, phencyclidine (PCP) derivatives

## Abstract

The dissociative anesthetic phencyclidine (PCP) and PCP derivatives, including 4′-F-PCP, are illegally sold and abused worldwide for recreational and non-medical uses. The psychopharmacological properties and abuse potential of 4′-F-PCP have not been fully characterized. In this study, we evaluated the psychomotor, rewarding, and reinforcing properties of 4′-F-PCP using the open-field test, conditioned place preference (CPP), and self-administration paradigms in rodents. Using Western immunoblotting, we also investigated the expression of dopamine (DA)-related proteins and DA-receptor-mediated downstream signaling cascades in the nucleus accumbens (NAc) of 4′-F-PCP-self-administering rats. Intraperitoneal administration of 10 mg/kg 4′-F-PCP significantly increased locomotor and rearing activities and increased CPP in mice. Intravenous administration of 1.0 mg/kg/infusion of 4′-F-PCP significantly enhanced self-administration during a 2 h session under fixed ratio schedules, showed a higher breakpoint during a 6 h session under progressive ratio schedules of reinforcement, and significantly altered the expression of DA transporter and DA D1 receptor in the NAc of rats self-administering 1.0 mg/kg 4′-F-PCP. Additionally, the expression of phosphorylated (p) ERK, pCREB, c-Fos, and FosB/ΔFosB in the NAc was significantly enhanced by 1.0 mg/kg 4′-F-PCP self-administration. Taken together, these findings suggest that 4′-F-PCP has a high potential for abuse, given its robust psychomotor, rewarding, and reinforcing properties via activation of DAergic neurotransmission and the downstream signaling pathways in the NAc.

## 1. Introduction

Phencyclidine (PCP) and PCP derivatives are dissociative anesthetics of the arylcyclohexylamine class and they are used clinically in animals and humans as general anesthetics [1,2]. Like other arylcyclohexylamines, PCP and PCP derivatives act as potent *N*-methyl-d-aspartate glutamate receptor (NMDAR) inhibitors [3,4], but they also work as a dopamine (DA) reuptake inhibitor [5,6]. The latter effect of PCP and PCP derivatives contributes to increases of synaptic DA levels in the medial frontal cortex and striatum [5,6], leading to a euphoric state. Due to the mind-altering effects of PCP and PCP derivatives, they have been sold for recreational and non-medical uses in illicit markets [2,7]. In addition, the recreational and non-medical uses of PCP and PCP derivatives have emerged as a major problem because they cause severe adverse effects including abuse, trance-like ecstatic states, hallucinations, and violent behavior in humans [2,8]. For these reasons, most countries control the use of PCP and PCP derivatives based on their abuse potential and hallucinogenic effects in humans [1].

In order to circumvent legal restrictions for the abuse of PCP and PCP derivatives, a variety of novel PCP derivatives such as 4-methoxyphencyclidine (4-MeO-PCP) and 3-methoxyphencyclidine (3-MeO-PCP) have been newly synthesized and sold on global illicit drug markets [7,8]. Based on structure–activity relationship studies, these novel PCP derivatives which are structurally similar to PCP also have the potential to cause neurobiological and psychopharmacological effects in vitro and in vivo [9,10]. Addition of fluorine (a chemical element) to ring structures of drugs can influence the metabolism and distribution of the original drug molecules in the body and can dramatically change their biological activities [11]. Recently, a study demonstrated that novel fluorinated PCP derivatives also have a high binding affinity to NMDAR in vitro and pharmacological efficacy in vivo [12]. These findings suggest that fluorinated PCP derivatives may have the potential to induce euphoric effects and they could be illegally abused like other PCP derivatives. A kind of novel fluorinated PCP derivative, 1-(1-(4-fluorophenyl)cyclohexyl)piperidine (4′-F-PCP), was first synthesized in 2002 [13]. Based on the pharmacological effects of other novel PCP derivatives, it seems that 4′-F-PCP has the potential to act as a bioactive molecule in the brain [11,12], but the neurobiological and psychopharmacological effects of 4′-F-PCP have not yet been fully characterized.

The rewarding and reinforcing properties of psychostimulants are closely associated with the mesolimbic dopaminergic system, projecting from the ventral tegmental area (VTA) to the nucleus accumbens (NAc). In addition, the rewarding and reinforcing properties of psychostimulants such as cocaine, methamphetamine, and PCP have been well demonstrated through various behavioral assessments including open-field test, conditioned place preference (CPP), and intravenous (i.v.) drug self-administration paradigms [14,15,16]. In previous studies, PCP and PCP derivatives produced robust increases in locomotor activity [17,18] and CPP in rodents [18,19,20]. Moreover, PCP and PCP derivatives were significantly self-administered in rodent and primate animal models [18,20,21,22]. These findings suggest that PCP and PCP derivatives not only cause psychotomimetic effects [2], but also produce psychological dependence with chronic use, due to their reward potential. However, there is currently no scientific evidence for the abuse potential of 4′-F-PCP.

Therefore, in this study, we demonstrated the abuse potential of 4′-F-PCP using multiple behavioral assessments and the mechanisms underlying addictive behaviors. We performed the open-field test to determine the psychomotor effects of 4′-F-PCP and the CPP test to examine the rewarding properties of 4′-F-PCP in mice. In addition, to determine the reinforcing effects of 4′-F-PCP, we performed self-administration studies under fixed ratio (FR) and progressive ratio (PR) schedules of reinforcement in rats. Finally, we evaluated the expression of DA-related proteins and extracellular signal-regulated kinase (ERK), cyclic AMP response-element binding protein (CREB), and Fos-family proteins in the NAc, a primary brain region mediating reward or reinforcing behavior, of 4′-F-PCP-self-administered rats.

## 2. Results

### 2.1. 4′-F-PCP Increased Locomotor and Rotational Activities in Mice

**Figure 1:**
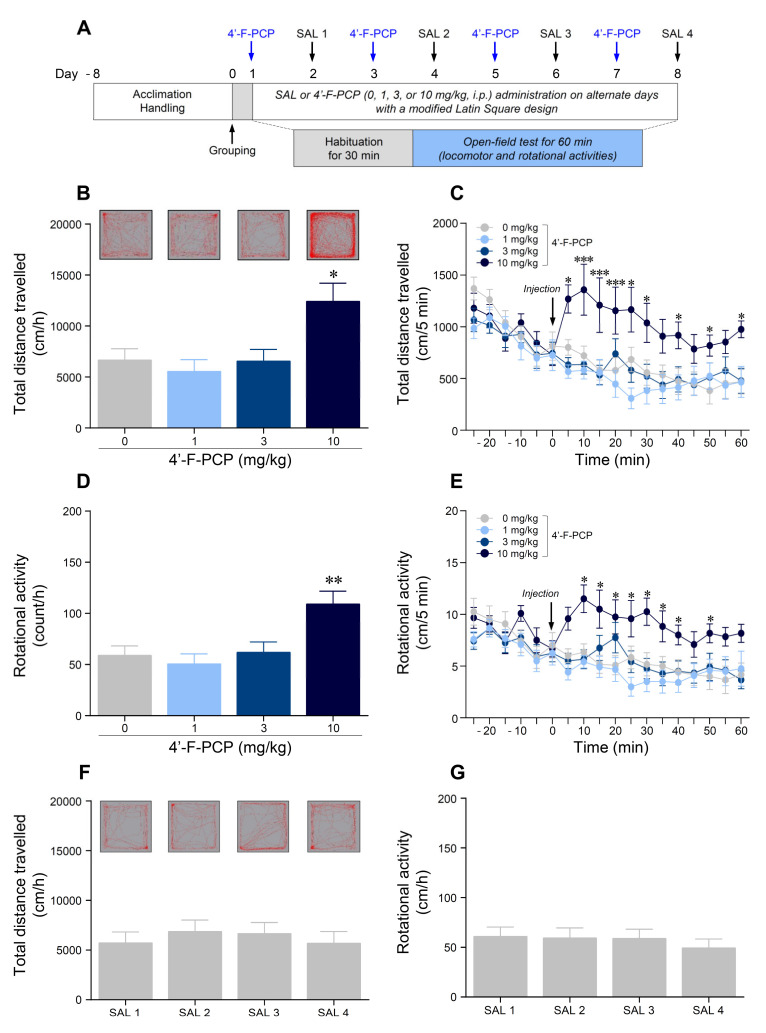
Effects of 1-(1-(4-fluorophenyl)cyclohexyl)piperidine (4′-F-PCP) on locomotor and rotational activities in mice. (**A**) Experimental timeline for open-field test of 4′-F-PCP. (**B**,**C**) Representative locomotion tracking patterns (red lines in each gray rectangle) and total distance travelled over 60 min, and temporal changes in distance travelled after administration of 4′-F-PCP (0, 1, 3, or 10 mg/kg, i.p.). (**D**,**E**) Total rotational activity over 60 min and temporal changes in rotational activity after administration of 4′-F-PCP (0, 1, 3, or 10 mg/kg, i.p.). (**F**,**G**) Representative locomotion tracking patterns with total distance travelled and rotational activity for 60 min after the administration of saline on the other alternate days. Asterisk *, **, and *** indicates *p* < 0.05, *p* < 0.01, and *p* < 0.001, respectively, vs. 0 mg/kg 4′-F-PCP group. SAL: saline. *n* = 12 per group.

As acute exposure to synthetic PCP derivatives produced a significant increase in psychomotor activity [18], we first performed the open-field test to determine whether 4′-F-PCP produces psychomotor hyperactivity. The timeline for the open-field test after 4′-F-PCP administration on the alternate days is illustrated in Figure 1A. The results showed that 10 mg/kg 4′-F-PCP administration, but not 1 or 3 mg/kg 4′-F-PCP, significantly increased locomotor activity (*F*_(3,34)_ = 5.68, *p* < 0.01; 10 mg/kg 4′-F-PCP: *p* < 0.05; Figure 1B) and rotational activity (*F*_(3,44)_ = 6.45, *p* < 0.001; 10 mg/kg 4′-F-PCP: *p* < 0.01; Figure 1D) compared to the saline control group. Similarly, in temporal analysis, 10 mg/kg 4′-F-PCP administration significantly increased locomotor activity (Time: *F*_(17,187)_ = 16.36, *p* < 0.001; Treatment: *F*_(3,33)_ = 11.27, *p* < 0.001; Interaction: *F*_(51,561)_ = 2.96, *p* < 0.001; Figure 1C) and rotational activity (Time: *F*_(17,187)_ = 7.85, *p* < 0.001; Treatment: *F*_(3,33)_ = 14.88, *p* < 0.001; Interaction: *F*_(51,561)_ = 2.07, *p* < 0.001; Figure 1E) in a time-dependent manner compared to the saline control group. However, there was no significant difference between clockwise and counterclockwise rotational propensities in the 10 mg/kg 4′-F-PCP-treated group. In addition, there was no significant difference in changes of locomotor activity (*F*_(3,44)_ = 0.30, *p* = 0.82) or rotational activity (*F*_(3,44)_ = 0.31, *p* = 0.81) among the other alternate days with saline administration (Day 2, 4, 6, and 8; Figure 1F,G).

### 2.2. 4′-F-PCP Induced Conditioned Place Preference in Mice

**Figure 2:**
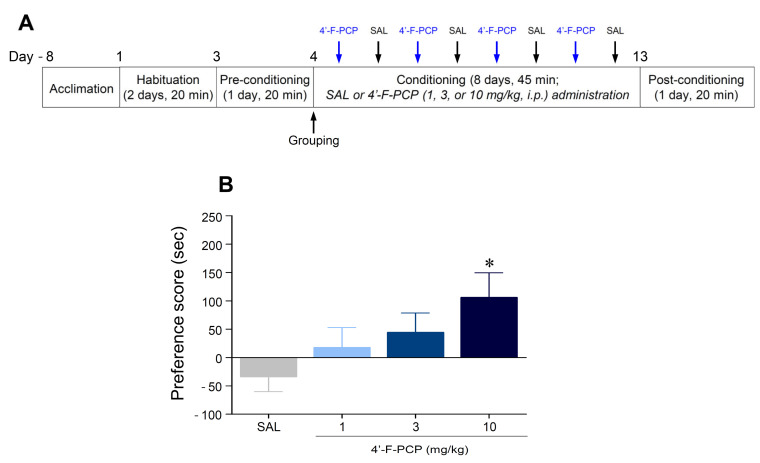
Effect of 4′-F-PCP on conditioned place preference (CPP) in mice. (**A**) Experimental timeline for CPP test of 4′-F-PCP. (**B**) Effect of 4′-F-PCP (1, 3, or 10 mg/kg, i.p.) on place preference. Asterisk * indicates *p* < 0.05 vs. saline-conditioned group. SAL: saline. *n* = 12 per group.

We applied the CPP paradigm to determine whether conditioning with 4′-F-PCP produces place preference in mice. The timeline for the CPP experiment is illustrated in Figure 2A. The results showed that the mice conditioned with 10 mg/kg 4′-F-PCP displayed a significant increase in place preference compared to the saline control group (*F*_(3,44)_ = 2.85, *p* < 0.05; 10 mg/kg 4′-F-PCP: *p* < 0.05; Figure 2B).

### 2.3. 4′-F-PCP Induced Self-Administration under Fixed Ratio Schedule in Rats

**Figure 3:**
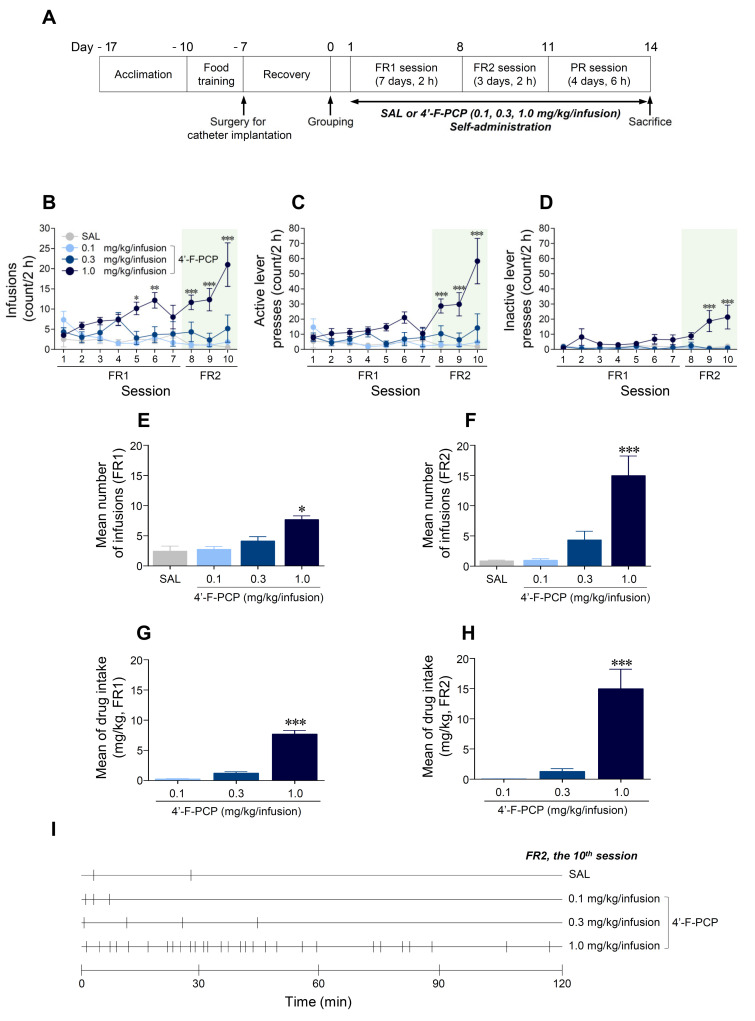
Effects of 4′-F-PCP on self-administration under fixed ratio (FR) schedule in rats. (**A**) Experimental timeline for 4′-F-PCP self-administration under FR and progressive ratio (PR) schedules of reinforcement. (**B**–**D**) Temporal analysis of mean number of infusions, active lever presses, and inactive lever presses during a 2 h session under FR1 and FR2 schedules. (**E**,**F**) Mean of total number of infusions for a 2 h session under FR1 and FR2 schedules. Asterisks *, **, and *** indicate *p* < 0.05, *p* < 0.01, and *p* < 0.001, respectively, vs. saline self-administered group. (**G**,**H**) Mean of intake amount of 4′-F-PCP for a 2 h session under FR1 and FR2. Asterisks *** indicate *p* < 0.001 vs. 0.1 and 0.3 mg/kg/infusion 4′-F-PCP group. (**I**) Representative hatchmarks indicate the infusion patterns of saline and 4′-F-PCP at the final self-administration session of FR2 schedules. SAL: saline. *n* = 6 per group.

We applied the self-administration paradigm under FR and PR schedules of reinforcement to determine whether 4′-F-PCP produces reinforcing effects in rats. The timeline for self-administration is illustrated in Figure 3A. Under FR schedules, the results showed that 1.0 mg/kg/infusion 4′-F-PCP-self-administered group significantly increased infusions (Session: *F*_(9,45)_ = 1.36, *p* = 0.23; Treatment: *F*_(3,15)_ = 15.67, *p* < 0.001; Interaction: *F*_(27,135)_ = 3.47, *p* < 0.001) and active lever-pressing responses (Session: *F*_(9,45)_ = 3.80, *p* < 0.01; Treatment: *F*_(3,15)_ = 16.00, *p* < 0.001; Interaction: *F*_(27,135)_ = 4.51, *p* < 0.001) compared to the saline control group (Figure 3B,C). Inactive lever-pressing responses at 1.0 mg/kg/infusion 4′-F-PCP group were significantly increased under FR2 schedules, but not FR1 schedules, compared to the saline group (Session: *F*_(9,45)_ = 2.60, *p* < 0.05; Treatment: *F*_(3,15)_ = 21.79, *p* < 0.001; Interaction: *F*_(27,135)_ = 2.54, *p* < 0.001; Figure 3D). To identify lever preference during the FR2 schedules, we performed a temporal analysis of the active and inactive lever-pressing responses at 1.0 mg/kg/infusion 4′-F-PCP group. The results showed that active lever-pressing responses were higher than inactive lever-pressing responses during the FR2 schedules (Supplementary Figure S1). 

In advanced analysis, the 1.0 mg/kg/infusion 4′-F-PCP group significantly increased the mean number of infusions under FR1 (*F*_(3,20)_ = 13.44, *p* < 0.001; 1.0 mg/kg/infusion 4′-F-PCP: *p* < 0.001; Figure 3E) and FR2 schedules (*F*_(3,20)_ = 14.23, *p* < 0.001; 1.0 mg/kg/infusion 4′-F-PCP: *p* < 0.001; Figure 3F,I) compared to the saline control group. Similarly, the results showed that amount of drug intake at 1.0 mg/kg/infusion 4′-F-PCP group was significantly increased under FR1 (*F*_(2,15)_ = 123.80, *p* < 0.001; 1.0 mg/kg/infusion 4′-F-PCP: *p* < 0.001; Figure 3G) and FR2 schedules (*F*_(2,15)_ = 19.46, *p* < 0.001; 1.0 mg/kg/infusion 4′-F-PCP: *p* < 0.001; Figure 3H) compared to the 0.1 and 0.3 mg/kg/infusion 4′-F-PCP groups.

### 2.4. 4′-F-PCP Produced Higher Breakpoint in Self-Administration under Progressive Ratio Schedule in Rats

**Figure 4:**
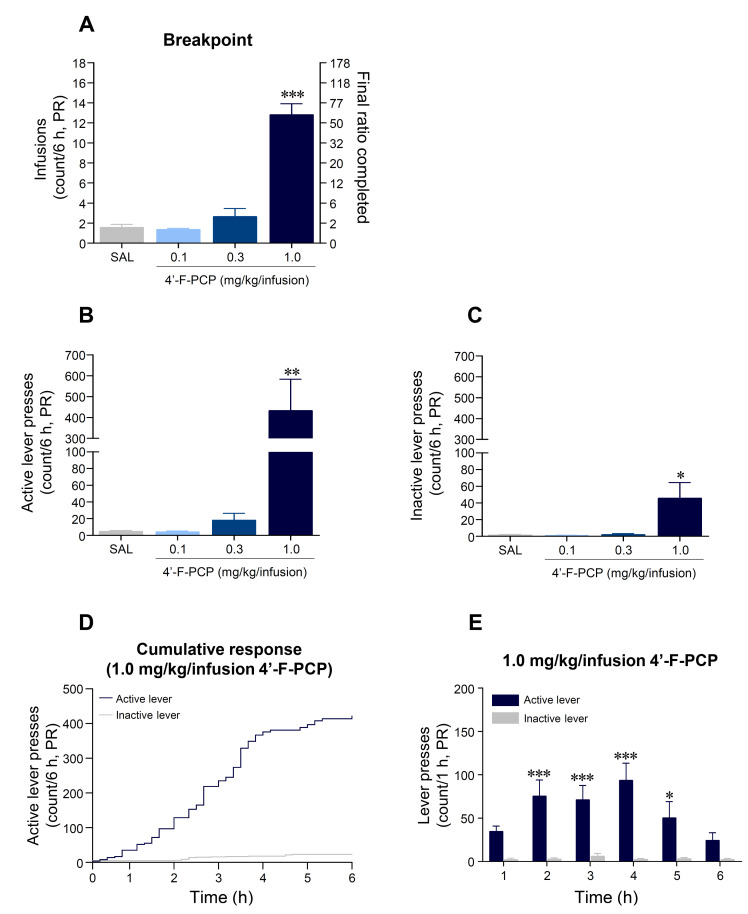
The reinforcing strength of 4′-F-PCP self-administration under the progressive ratio (PR) schedules in rats. (**A**) Breakpoint of saline and 4′-F-PCP (0.1, 0.3, or 1.0 mg/kg/infusion) during 6 h under the PR sessions (Day 11–14). Left y-axis and right y-axis indicate the mean of drug infusions and final ratio completed to access saline or 4′-F-PCP for 4 days of PR schedule, respectively. (**B**,**C**) Mean number of active and inactive lever-pressing responses of saline or 4′-F-PCP-self-administration under the PR schedules. (**D**) Representative plots for cumulative active and inactive lever responses at 1.0 mg/kg/infusion 4′-F-PCP-self-administering rats during the final session of PR schedule. (**E**) Temporal analysis of active and inactive lever responses at the final session of PR schedule of 1.0 mg/kg/infusion 4′-F-PCP-self-administered rats. Asterisk *, **, and *** indicate *p* < 0.05, *p* < 0.01, and *p* < 0.001, respectively, vs. inactive lever. SAL: saline. *n* = 6 per group.

Under the PR schedules of reinforcement, the mean breakpoint at 1.0 mg/kg/infusion 4′-F-PCP-self-administered group was significantly increased compared to the saline control group (*F*_(3,20)_ = 64.34, *p* < 0.001; 1.0 mg/kg/infusion 4′-F-PCP: *p* < 0.001; Figure 4A). Similarly, our results showed that the mean of active lever-pressing responses at 1.0 mg/kg/infusion 4′-F-PCP group was significantly increased (*F*_(3,20)_ = 8.01, *p* < 0.01; 1.0 mg/kg/infusion 4′-F-PCP: *p* < 0.01) as well as inactive lever-pressing responses (*F*_(3,20)_ = 5.70, *p* < 0.05; 1.0 mg/kg/infusion 4′-F-PCP: *p* < 0.05) compared to saline control group (Figure 4B,C). In a temporal analysis of cumulative lever-pressing responses at 1.0 mg/kg/infusion 4′-F-PCP-self-administered group, the mean of active lever-pressing responses was significantly higher than the mean of inactive lever-pressing responses during the final session of PR schedule (Time: *F*_(5,60)_ = 2.72, *p* < 0.05; Lever: *F*_(5,60)_ = 2.92, *p* < 0.05; Interaction: *F*_(1,6)_ = 73.68, *p* < 0.001; Figure 4D,E).

### 2.5. 4′-F-PCP Self-Administration Altered Expression of Dopamine-Related Proteins in the Nucleus Accumbens

**Figure 5:**
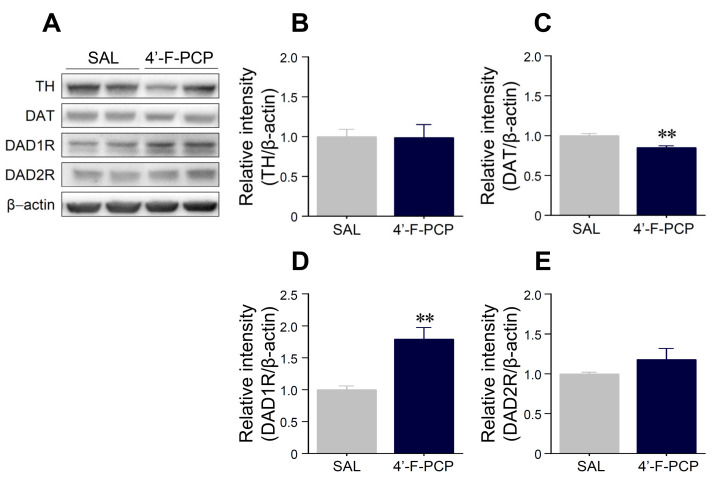
Effect of 4′-F-PCP on the immunoreactivities of dopamine-related proteins in the nucleus accumbens (NAc) of 4′-F-PCP-self-administered rats under the PR schedules. (**A**–**E**) Immunoreactivities of TH, DAT, DAD1R, and DAD2R proteins in the NAc after saline or 1.0 mg/kg/infusion 4′-F-PCP self-administration under the PR schedules of reinforcement. Asterisks ** indicate *p* < 0.01 vs. saline controls. SAL: saline. *n* = 4–6 per group.

Since drug-induced rewarding and reinforcing effects are closely related to modulation of DAergic neurotransmission in the reward system [23,24,25], we determined whether 4-F-PCP alters the expression of DA-related proteins such as tyrosine hydroxylase (TH), dopamine transporter (DAT), DA D1 receptor (DAD1R), and DA D2 receptor (DAD2R) in the NAc of 4-F-PCP-self-administered rats after the final PR schedule of reinforcement. The results showed that the 4-F-PCP-self-administration group had significantly decreased DAT (*t*_(8)_ = 4.26, *p* < 0.01) and increased DAD1R immunoreactivities (*t*_(8)_ = 3.49, *p* < 0.01) in the NAc compared to saline control group (Figure 5A,C,D). However, there was no difference in the immunoreactivities of TH and DAD2R in the NAc of 4′-F-PCP-self-administered rats compared to the saline control group (Figure 5A,B,E).

### 2.6. 4′-F-PCP Self-Administration Altered Expression of pERK, pCREB, c-Fos, and FosB/ΔFosB in the Nucleus Accumbens

**Figure 6:**
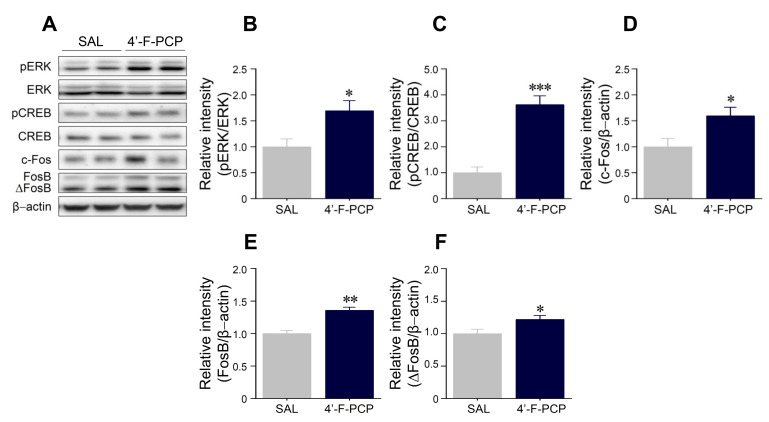
Effect of 4′-F-PCP on the immunoreactivities of pERK, pCREB, c-Fos, and FosB/ΔFosB proteins in the NAc of 4′-F-PCP-self-administered rats under the PR schedule. (**A**–**F**) Immunoreactivities of pERK, pCREB, c-Fos, and FosB/ΔFosB proteins in the NAc after saline or 1.0 mg/kg/infusion 4′-F-PCP self-administration under the PR schedules of reinforcement. Asterisks *, **, and *** indicate *p* < 0.05, *p* < 0.01, and *p* < 0.001, respectively, vs. saline controls. SAL: saline. *n* = 4–6 per group.

Since DAD1R levels were altered at 1.0 mg/kg/infusion 4′-F-PCP-self-administered rats, we investigated whether 1.0 mg/kg/infusion 4′-F-PCP alters DAD1R-mediated downstream signaling cascades in the NAc of administered rats. As shown in Figure 6, the immunoreactivities of phosphorylated (p) ERK (*t*_(8)_ *=* 2.55, *p* < 0.05) and pCREB (*t*_(8)_ = 5.76, *p* < 0.001) in the NAc of 1.0 mg/kg/infusion 4′-F-PCP-self-administered rats were also significantly increased compared to saline control groups (Figure 6A–C) without changes of the immunoreactivities of total ERK and CREB (Figure 6A). Additionally, 4′-F-PCP significantly increased the immunoreactivity of c-Fos (*t*_(8)_ = 2.43, *p* < 0.05), FosB (*t*_(8)_ = 5.02, *p* < 0.01), and ΔFosB *(t*_(8)_ = 2.36, *p* < 0.05) in the NAc of self-administered rats compared to the saline control group (Figure 6A,D,F).

## 3. Discussion

In the present study, we demonstrated for the first time the abuse potential of 4′-F-PCP. The results showed that 4′-F-PCP significantly (1) increased locomotor and rotational activities in mice; (2) produced a drug-paired place preference in mice; (3) increased number of active lever-pressing responses and drug infusions under the FR and PR schedules of reinforcement in rats; (4) decreased the expression of DAT in the NAc of 4′-F-PCP-self-administered rats; (5) enhanced the expression of DAD1R in the NAc of 4′-F-PCP-self-administered rats; and (6) enhanced the expression of pERK, pCREB, c-Fos, FosB, and ΔFosB in the NAc of 4′-F-PCP-self-administered rats. Taken together, our findings suggest that 4′-F-PCP has an abuse potential, given its robust psychomotor, rewarding, and reinforcing properties, in part via the activation of DAergic neurotransmission and its downstream signaling pathways in the reward system of rodents.

Drug-induced increases in psychomotor activities are closely related to hyperstimulation of the mesolimbic DAergic reward system [26]. Previous studies reported that administration of PCP or PCP derivatives increased locomotor and rotational activities in a dose-dependent manner [17,18,27,28], and its psychomotor effects were blocked by antagonism of DAD1R activity [17]. Similarly, administration of ketamine or methoxetamine, a kind of PCP-like substance, also increased psychomotor responses in a dose-dependent manner in rodents [29,30,31]. Consistent with these findings, our results demonstrated that 10 mg/kg 4′-F-PCP administration, but not 1 or 3 mg/kg, significantly increased locomotor and rotational activities in mice. Taken together, these findings suggest that 4′-F-PCP is a potent psychoactive drug and produces psychobehavioral effects in a dose-dependent manner.

The CPP is another classic animal model used to evaluate the rewarding effect of contextual stimuli associated with exposure to addictive drugs [14,15]. Previous studies demonstrated that repeated administration of PCP or PCP derivatives produced a positive preference [18,19,20]. Consistent with these findings, our results demonstrated that 4′-F-PCP at a dose of 10 mg/kg produced a significant increase in preference in mice. Taken together, these findings suggest that 4′-F-PCP has a rewarding effect like PCP and other PCP derivatives. It is well-known that the PCP-induced rewarding effect is closely related to activation of the mesolimbic DAergic system [19,31]. The structure–activity relationship studies demonstrated that PCP derivatives can act as DA reuptake inhibitors due to their structural similarity to PCP, thereby increasing extracellular DA levels in the brain [9,10,19]. For example, Abiero et al. demonstrated that 4-MeO-PCP and 3-MeO-PCMo, which are structurally similar to PCP, increased DA concentration in the NAc of mice [18]. Based on these findings, we speculate that 4′-F-PCP enhances psychomotor activities and produces the preference via increased DA levels in the NAc.

I.v. self-administration in rodents is a useful model for predicting the abuse liability of novel drugs in humans [15]. Previous preclinical studies have demonstrated a dose–response relationship for the rate of PCP self-administration and level of drug intake in rats and monkeys under FR schedules of reinforcement [21,22]. In addition, PCP derivatives such as 4-MeO-PCP, 3-MeO-PCMo, and ketamine were also self-administered in rats via activation of DAergic neurotransmission in the NAc [18]. Consistent with these findings, our results demonstrated that 4′-F-PCP at a high dose of 1.0 mg/kg/infusion, but not 0.1 and 0.3 mg/kg/infusion, significantly increased the active lever-pressing responses for drug taking under FR1 and FR2 schedules of reinforcement. Taken together, these findings suggest that only a high dose of 4′-F-PCP can pharmacologically act as a positive reinforcer in the brain reward system, producing reinforcing effects in rats.

The progressive schedule of reinforcement in self-administration paradigms has been used to directly measure the strength of reinforcement (i.e., how hard the animal will work) of psychostimulants with abuse potential by increasing the response requirement for successive reinforcements [32]. Previous self-administration studies demonstrated that various psychoactive drugs such as cocaine, PCP, and PCP derivatives showed a positive dose–response relationship under a PR schedule of reinforcement [18,33,34]. Consistent with these findings, our results demonstrate that 1.0 mg/kg/infusion 4′-F-PCP, but not 0.1 or 0.3 mg/kg/infusion, self-administration under the PR schedules following the FR schedules significantly increased drug-taking behavior. The breakpoint of 1.0 mg/kg/infusion 4′-F-PCP was similar to those of 0.3 mg/kg/infusion 4-MeO-PCP and 3-MeO-PCMo, which are new PCP-derivative dissociative drugs [18]. Moreover, the reinforcing efficacy of PCP derivatives varies from derivative to derivative [35]. In particular, the addition of fluorine to the ring structure of a drug alters its psychopharmacological effects and biological activities [11,36]. Based on these results, it could be speculated that a fluorine substitution in PCP also produces a relatively strong reinforcement, like other PCP derivatives, in a dose-dependent manner.

In general, a positive reinforcer (i.e., drugs) is provided to animals when they successfully accessed the drug-paired lever (active lever) in self-administration studies [15]. However, unexpectedly, we found that number of inactive lever responses (not paired with drug) were also significantly increased compared to that of the saline control group during the FR2 (9th and 10th sessions) and PR schedules of reinforcement at 1.0 mg/kg/infusion 4′-F-PCP-self-administered rats. However, upon advanced analysis, the results demonstrated that the response ratio of active lever presses was over 74.1% during the FR2 schedules (8th session: 76.4%; 9th session: 68.1%; 10th session: 77.6%) and over 90.7% during the PR schedules. Based on the results of lever preference in the 1.0 mg/kg/infusion 4′-F-PCP group, the response to the drug-paired lever was well-reinforced in the 4′-F-PCP-self-administered rats. In reference to previous studies, administration of PCP or PCP derivatives commonly induces hallucinogenic effects and loss of balance with staggering behavior [1,37]. Consistently, 4′-F-PCP (1.0 mg/kg/infusion) self-administered rats in this study showed staggering behavior during the FR2 and PR schedules of reinforcement. Taken together, it could be thought that non-specific responses to inactive lever were due to staggering behavior caused by 4′-F-PCP self-administration.

TH and DAT are well-known as modulators of DA concentrations in the reward system. TH, a rate-limiting enzyme, is involved in synthesis of DA, and DAT controls DA concentrations in the synaptic cleft and neurotransmission via reuptake of DA into the presynaptic terminals [38,39]. Our results demonstrated that 4′-F-PCP self-administration decreased DAT expression in the NAc, but the expression of TH was not altered. Based on these findings, we speculate that 4′-F-PCP produces a reinforcing effect by inhibiting DA reuptake in DAergic terminals of the NAc, rather than increases in DA synthesis [38]. However, acute administration of new synthetic PCP derivatives (4-MeO-PCP and 3-MeO-PCMo) or ketamine did not alter DAT expression in the NAc of mice [18], suggesting that the expression and function of DAT can vary depending on experimental paradigm (type of exposed PCP derivatives, route of administration, dosing period, etc.). Moreover, a previous study reported that exposure to psychoactive drugs such as PCP derivatives and ketamine significantly increased DAD1R expression and decreased DAD2R expression in the NAc [18]. Accordingly, we found that the DAD1R expression was significantly enhanced in the NAc of 4′-F-PCP-self-administered rats under the PR schedules of reinforcement. However, 4′-F-PCP did not alter DAD2R expression in the NAc. Taken together, these findings suggest that the stimulation of DAD1R rather than DAD2R may play an important role in the psychobehavioral properties of 4′-F-PCP.

ERK, a member of MAP kinase family, is a well-known downstream target of DAD1R-mediated signaling cascades involved in reward and behavioral changes due to drugs such as cocaine, amphetamine, and methamphetamine [40]. ERK phosphorylation by external stimuli including drug exposure can activate transcription factors such as pCREB, c-Fos, and FosB/ΔFosB in the NAc [40]. The activation and accumulation of these transcription factors in the NAc are implicated in neuroadaptation to drug abuse [41]. These findings suggest that the activation of ERK, CREB, c-Fos, and FosB/ΔFosB have been closely linked to drug-induced reward and addiction [40,41,42,43]. Consistent with these findings, our results demonstrated that 4′-F-PCP significantly enhanced pERK, pCREB, c-Fos, and FosB/ΔFosB levels in the NAc of self-administered rats. Taken together, these findings suggest that activation of ERK–CREB pathway and c-Fos, and FosB/ΔFosB accumulations in the NAc reflect 4′-F-PCP-induced neuroadaptations, which may contribute to the rewarding and reinforcing effects of 4′-F-PCP, leading to abuse.

However, the present study had a limitation in that we only evaluated the 4′-F-PCP-induced alternations in DAergic neurotransmission of the NAc. Others have demonstrated previously that PCP derivatives can affect glutamatergic functions by acting as potent NMDAR inhibitors [3,4]; the altered glutamate system also influences dopaminergic neurotransmission in the reward system [23,44]. Additionally, other brain regions such as the dorsal striatum, prefrontal cortex, and hippocampus also play important roles in the development of substance abuse [23]. Thus, further studies are needed to investigate the brain-region-specific involvement of dopaminergic and glutamatergic neurotransmission in 4′-F-PCP abuse.

In summary, we demonstrated that 4′-F-PCP produces psychomotor hyperactivity, place preference, self-administration, and altered expression of DA-related proteins (DAT and DAD1R) and pERK, pCREB, c-Fos, and FosB/ΔFosB in the NAc. In conclusion, 4′-F-PCP produces psychopharmacological properties via activation of DAD1R-mediated neurotransmission in the reward system, providing compelling pre-clinical evidence of its abuse potential in humans. Moreover, these findings have important implications for the development of appropriate drug legislative measures and for predicting the potential for abuse of new synthetic PCP derivatives.

## 4. Materials and Methods

### 4.1. Animals

Male C57BL/6 mice (18–25 g) for open-field and CPP tests and male Sprague–Dawley rats (235–275 g) for self-administration tests were obtained from Orient Bio. Inc. (Seongnam, South Korea). Animals were maintained in a temperature- (23 ± 3 °C) and humidity-controlled (30–70%) facility under a regular 12 h light–dark cycle (lights on at 8:00 AM). Mice were housed in groups of four animals and rats were housed individually. Food and water were provided ad libitum, except during food training periods of self-administration. During food training periods, rats were food-restricted to 13–15 g/day to increase the probability of lever responding. All animal experimental measurements were conducted in a quiet room to minimize environmental stress during the light cycle. All experimental procedures were approved by the Institutional Animal Care and Use Committee at the Korea Institute of Toxicology (Approval No. KIT-1804-0164, 26/04/2018) and were conducted in accordance with the provisions of the National Institutes of Health Guide for the Care and Use of Laboratory Animals.

### 4.2. Drugs

4′-F-PCP was synthesized and provided by the Laboratory of Medicinal Chemistry of Kyunghee University (Seoul, South Korea) as described previously [13]. The chemical structure of 4′-F-PCP is represented in Supplementary Figure S2. 4′-F-PCP was dissolved in 0.9% sterile physiological saline. The dose determinations for each behavioral test in this study was based on previous studies of the abuse potential of PCP derivatives [18,19,20]. All drug solutions were prepared immediately prior to the beginning of each experiment. For self-administration studies, a working solution of 4′-F-PCP was filtered through a syringe-mounted 0.22 µm Minisart^®^ Syringe Filter (Sartorius Stedim Biotech, Goettingen, Germany) immediately before use.

### 4.3. Open-Field Test

The open-field test was performed as described previously [16]. Mice were acclimated to the open-field arena in an illuminated and sound-attenuated cubicle for at least 6 days (for 30 min/day). Mice received intraperitoneal (i.p.) injections of 4′-F-PCP (0, 1, 3, or 10 mg/kg, 10 mL/kg) on the alternate days under a modified Latin squared design (Figure 2A and Supplementary Figure S3) [45]. To test the accumulated effects of the remaining 4′-F-PCP in the body due to its long half-life and long-lasting action [17,30], we added a saline intercalate administration between the alternating administrations of 4′-F-PCP in this study. Locomotor activity (total distance travelled, centimeters) and rotational activity (turning around, count) were recorded using a computer-based monochrome/near-infrared video camera. On the test day, mice were placed in the open-field arena and basal activity was measured for 30 min. After recording basal activity, mice received an injection of saline or 4′-F-PCP and behavioral activities were then recorded for an additional 1 h. Changes in locomotor activity and rotational activity were quantified using a computer-based video tracking system (Ethovision XT14, version 14.0.1322, Noldus, Wageningen, The Netherlands).

### 4.4. Conditioned Place Preference Paradigm

The CPP experiment was performed based on our previous study [16]. The CPP apparatus (MED-CPP-3013-2, Med Associates) consisted of two large compartments (17.4 cm × 12.7 cm × 12.7 cm) separated by a guillotine door. One compartment was a black room with a stainless steel grid floor consisting of rods (diameter: 3.2 mm) placed 7.9 mm apart. The other compartment was a white room with a 6.35 mm stainless steel mesh floor. The guillotine door that provided access was placed in the center of the two conditioning compartments. Each compartment had a Plexiglas top with controlled illumination (15–20 lux). The CPP paradigm was performed according to unbiased and counterbalanced subject assignment procedure. Briefly, the CPP test was composed of four different phases: (1) habituation phase (Day 1 and 2); (2) pre-conditioning phase (Day 3); (3) conditioning phase (Day 4–11); and (4) post-conditioning phase (Day 12) (Figure 2A). In the habituation phase, mice were placed in the CPP apparatus for 20 min with free access to both compartments. In the pre-conditioning phase, the time spent in each compartment was recorded for 20 min. These pre-conditioning data were used to classify the mice into groups that showed approximately equal preference for either side of the apparatus. The mice that stayed over 960 s on either side during the pre-conditioning test were excluded from this study. The cutoff value (>960 s) for biased preference was determined by our previous study [16]. During the conditioning phase, the mice were given an i.p. injection (10 mL/kg) of saline or 4′-F-PCP (1, 3, or 10 mg/kg, 10 mL/kg) on alternate days (4′-F-PCP: Day 4, 6, 8, and 10; saline: Day 5, 7, 9, and 11). Each mouse was then confined to one of the compartments after saline injection, or to the other compartments after 4′-F-PCP injection for 45 min (counterbalanced). In the post-conditioning phase, each mouse was allowed to roam freely between the both sides of the apparatus for 20 min. A preference score was determined by calculating the difference between the times spent in the drug-paired side of the apparatus during the pre- versus post-conditioning phases, as described previously [16].

### 4.5. Food Training and Catheter Implantation Surgery for Self-Administration

The food training and catheter implantation surgery were performed as described previously [16]. In brief, rats were trained to press a lever for 45 mg food pellets (BioServ, Frenchtown, NJ, USA) under a continuous FR1 schedule of reinforcement during 1 h sessions. After the acquisition criterion (obtained more than 80 food pellets/1 h, three consecutive days), rats were anesthetized with 2–4% isoflurane, and a chronic indwelling jugular catheter (inner diameter: 0.02 inch, outer diameter: 0.03 inch; Dow Corning, Midland, MI, USA) was inserted into the right jugular vein and secured to the muscle around the jugular vein with Mersilene surgical mesh (Ethicon Inc., Somerville, NJ, USA). The distal end of the catheter was threaded subcutaneously and connected to a 22 gauge stainless steel cannula (P1 Technologies, Roanoke, VA, USA) fixed to the head assembly with dental cement and secured with Prolene surgical mesh (Ethicon Inc.). After catheter implantation into the jugular vein, rats were given at least 7 days to recover prior to the self-administration test. During recovery, the catheter was flushed once daily with 0.2 mL of heparinized saline (30 IU/mL) including gentamicin sulfate (0.33 mg/mL) to prevent clotting and infection.

### 4.6. Drug Self-Administration

The drug self-administration test was performed as described previously [16]. After recovery, rats began 4′-F-PCP-self-administration test for 14 consecutive days under FR schedules of reinforcement (FR1: Day 1–7, FR2: Day 8–10, 2 h session) and then PR schedules of reinforcement (Day 11–14, 6 h session) (Figure 3A). During self-administration sessions, a response on the active lever (drug-paired lever) resulted in a 0.1 mL i.v. delivery of saline or 4′-F-PCP (0.1, 0.3, or 1.0 mg/kg/infusion) for 4.1 s. Each infusion was followed by an additional 20 s time-out period. During the time-out period, active lever responses were recorded, but did not result in infusion of drug. Inactive lever presses were also recorded, but had no consequence.

After 10 consecutive FR sessions, rats underwent daily 6 h self-administration session of PR schedules for 4 days to assess the reinforcing efficacy of 4′-F-PCP [16]. During the PR schedules, the number of lever presses required to obtain a single infusion of 4′-F-PCP was determined by the following equation: responses per drug delivery = [5e^(injection number × 0.2)^]−5 (i.e., 1, 2, 4, 6, 9, 12, 15, 20, 25, 32, 40, 50, 62, 77, and 95) [32]. The total lever presses and infusions were recorded throughout the session.

### 4.7. Western Immunoblotting Procedures

Western immunoblotting was performed as previously described [46]. After the final session of PR schedule on day 14, saline or 1.0 mg/kg/infusion 4′-F-PCP-self-administered rats were deeply anesthetized with 2–4% isoflurane, decapitated, and brains were rapidly removed. Tissue sections were serially cut using a stainless steel coronal brain matrix (Roboz Surgical Instrument Co., Inc., Gaithersburg, MD, USA) on ice, and the NAc was collected bilaterally. Brains were frozen in liquefied nitrogen and stored in a deep freezer until use. Tissues were transferred to mixture of RIPA buffer (Thermo Fisher Scientific, Waltham, MA, USA) containing protease and phosphatase inhibitor cocktails (Thermo Fisher Scientific), and were then homogenized and incubated for 1 h at 4 °C. After incubation, samples were centrifuged at 13,000 rpm for 30 min at 4 °C. The pellet, which primarily contained nuclei and large debris, was discarded and the supernatant was centrifuged again at 13,000 rpm for 30 min at 4 °C. The concentration of the solubilized proteins in the supernatant fraction was determined based on the bicinchoninic acid assay using a bicinchoninic acid assay kit (Thermo Fisher Scientific). The proteins in the supernatant fraction were resolved using 10% bisacrylamide gel electrophoresis, and the separated proteins were then transferred to a polyvinylidene fluoride membrane (Bio-Rad, Hercules, CA, USA). The membrane was treated with blocking buffer containing 5% bovine serum albumin in a mixture of Tris-buffered saline and 0.1% Tween-20 (TBST), and washed three times for 10 min each with TBST. After washing, the membrane was probed with a rabbit or a mouse primary antiserum for TH (1:1000; #2792S, Cell Signaling Technology, Danvers, MA, USA), DAT (1:1000; #ab111468, abcam, Cambridge, MA, USA), DAD1R (1:1000; #ab20066, abcam), DAD2R (1:1000; #sc-5303, Santa Cruz Biotechnology, Dallas, TX, USA), pERK (1:1000; #4377, Cell Signaling Technology), pCREB (1:1,000; #9198, Cell Signaling Technology), c-Fos (1:500; #sc-166940, Santa Cruz Biotechnology), and FosB/ΔFosB (1:1,000; #2251, Cell Signaling Technology) for 18 h at 4 °C on a shaker. The membrane was then re-washed three times and incubated with goat anti-rabbit IgG HRP- or goat anti-mouse IgG HRP-labeled secondary antiserum (Thermo Fisher Scientific) for 1 h at room temperature. Immunoreactive protein bands were detected with enhanced chemiluminescence reagents (Thermo Fisher Scientific) using Image Lab software (version 5.2.1, Bio-Rad). The membranes were re-probed using a rabbit primary antiserum against total ERK (1:1,000; #4695, Cell Signaling Technology), and CREB (1:1000; #9197, Cell Signaling Technology) after stripping the same membrane that was confirmed to contain the phosphorylated protein and re-probed for β-actin (1:2000; #A5316, Sigma-Aldrich, St. Louis, MO, USA) blot normalization. Immunoreactive protein bands on the membrane were semi-quantified using ImageJ software (version 1.52a, National Institutes of Health, USA).

### 4.8. Statistical Analyses

Statistical analyses were performed using GraphPad Prism 8 (version 8.0.2, GraphPad Software, La Jolla, CA, USA). Data are represented as mean ± standard error of mean (SEM). For Western immunoblotting data, statistical significance between the groups was determined by two-tailed unpaired t-tests. For behavioral data, Tukey’s post hoc test was used for all one-way analysis of variance (ANOVA) with repeated measures, and Bonferroni’s post hoc test was used for all repeated measures two-way ANOVA. The level of statistical significance was set at *p* < 0.05. Asterisks *, **, *** in figures represent significance levels *p* < 0.05, 0.01, and 0.001, respectively.

## Supplementary Materials

Supplementary materials can be found at https://www.mdpi.com/1422-0067/21/13/4631/s1. Figure S1. Lever presses and response ratio at 1.0 mg/kg/infusion 4’-F-PCP self-administered rats under FR2 schedules. (A–C) Temporal changes in active and inactive lever responses for 2 h during the FR2 schedules (the 8th–10th sessions) at 1.0 mg/kg/infusion 4’-F-PCP self-administered rats. (D–F) Temporal changes in response ratio to active or inactive lever during the FR2 schedules. The numbers in parentheses indicate the response ratio for each lever. n = 6 per group. Figure S2. (A) The chemical structures of phencyclidine (PCP) and (B) 1-(1-(4-fluorophenyl)cyclohexyl)piperidine (4’-F-PCP). Figure S3. Modified 4 × 4 Latin square design for open-field test.

## Abbreviations

3-MeO-PCP	3-Methoxyphencyclidine
4-MeO-PCP	4-Methoxyphencyclidine
4′-F-PCP	1-(1-(4-fluorophenyl)cyclohexyl)piperidine
CPP	Conditioned Place Preference
CREB	cyclic AMP Response-Element Binding Protein
DA	Dopamine
DAD1R	Dopamine D1 Receptor
DAD2R	Dopamine D2 Receptor
DAergic	Dopaminergic
DAT	Dopamine Transporter
ERK	Extracellular Signal-regulated Kinase
FR	Fixed Ratio
I.V.	intravenous
MAPK	Mitogen-activated Protein Kinase
NAc	Nucleus Accumbens
NMDAR	N-methyl-D-aspartate Glutamate Receptor
PCP	Phencyclidine
PR	Progressive Ratio
TH	Tyrosine Hydroxylase
VTA	Ventral Tegmental Area
