# Supplementary material for: The Abuse Potential of Novel Synthetic Phencyclidine Derivative 1-(1-(4-Fluorophenyl)Cyclohexyl)Piperidine (4′-F-PCP) in Rodents
Source: Int J Mol Sci. 2020 Jun 29;21(13):4631. doi: 10.3390/ijms21134631 (PMC7369973; doi:10.3390/ijms21134631)
Supplement: Supplementary file 1 [file ijms-21-04631-s001.pdf]

Supplementary

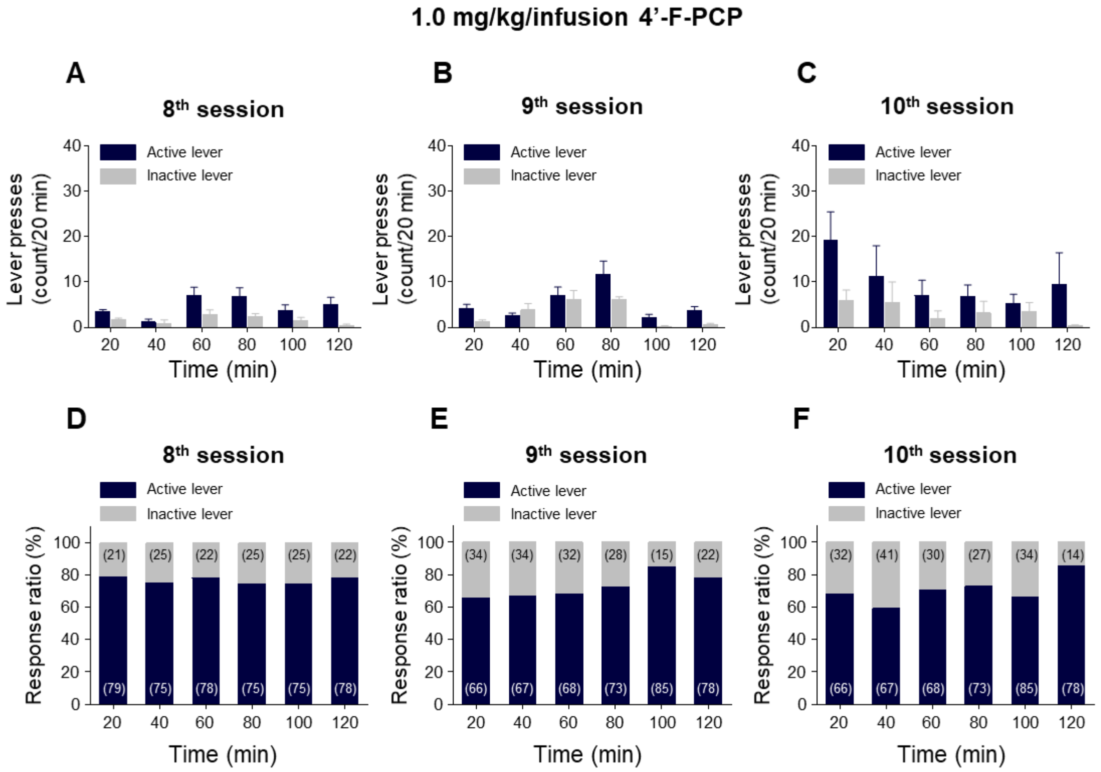

Figure S1. Lever presses and response ratio at 1.0 mg/kg/infusion 4′-F-PCP self-administered rats under FR2 schedules. (**A**–**C**) Temporal changes in active and inactive lever responses for 2 hr during the FR2 schedules (the 8th–10th sessions) at 1.0 mg/kg/infusion 4′-F-PCP self-administered rats. **(D**–**F)** Temporal changes in response ratio to active or inactive lever during the FR2 schedules. The numbers in parentheses indicate the response ratio for each lever. $n = 6$ per group.

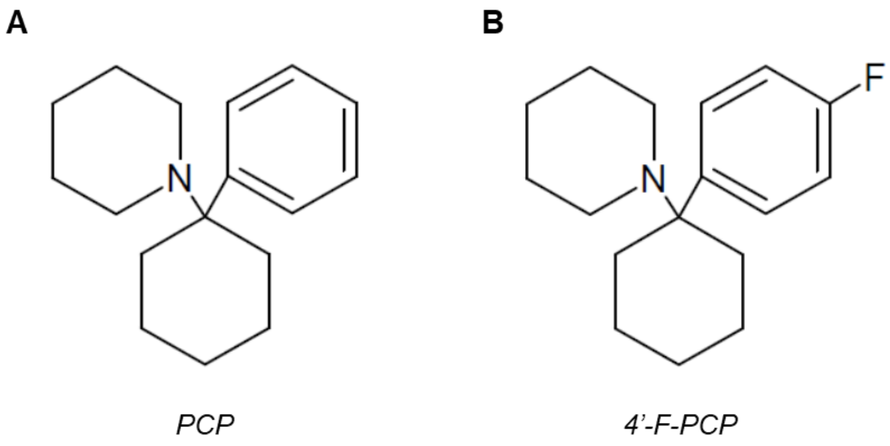

Figure S2. (**A**) The chemical structures of phencyclidine (PCP) and (**B**) 1-(1-(4-fluorophenyl)cyclohexyl)piperidine (4′-F-PCP).

Modified 4 x 4 Latin square design

Order	Group (mg/kg) A	B	C	D
1	10	SAL	3	1
2	SAL	10	1	3
3	3	1	SAL	10
4	1	3	10	SAL

Figure S3. Modified 4 × 4 Latin square design for open-field test.
